# *Cis-* and *Trans*-Regulatory Variations in the Domestication of the Chili Pepper Fruit

**DOI:** 10.1093/molbev/msaa027

**Published:** 2020-02-07

**Authors:** Erik Díaz-Valenzuela, Ruairidh H Sawers, Angélica Cibrián-Jaramillo

**Affiliations:** m1 Ecological and Evolutionary Genomics Laboratory, Unidad de Genomica Avanzada (Langebio), Irapuato, Guanajuato, México; m2 Department of Plant Science, The Pennsylvania State University, University Park State College, University Park, PA

**Keywords:** allele-specific expression, *Capsicum*, regulation, domestication

## Abstract

The process of domestication requires the rapid transformation of the wild morphology into the cultivated forms that humans select for. This process often takes place through changes in the regulation of genes, yet, there is no definite pattern on the role of *cis*- and *trans*-acting regulatory variations in the domestication of the fruit among crops. Using allele-specific expression and network analyses, we characterized the regulatory patterns and the inheritance of gene expression in wild and cultivated accessions of chili pepper, a crop with remarkable fruit morphological variation. We propose that gene expression differences associated to the cultivated form are best explained by *cis*-regulatory hubs acting through *trans-*regulatory cascades. We show that in cultivated chili, the expression of genes associated with fruit morphology is partially recessive with respect to those in the wild relative, consistent with the hybrid fruit phenotype. Decreased expression of fruit maturation and growth genes in cultivated chili suggest that selection for loss-of-function took place in its domestication. *Trans*-regulatory changes underlie the majority of the genes showing regulatory divergence and had larger effect sizes on gene expression than *cis*-regulatory variants. Network analysis of selected *cis*-regulated genes, including *ARP9* and *MED25*, indicated their interaction with many transcription factors involved in organ growth and fruit ripening. Differentially expressed genes linked to *cis*-regulatory variants and their interactions with downstream *trans*-acting genes have the potential to drive the morphological differences observed between wild and cultivated fruits and provide an attractive mechanism of morphological transformation during the domestication of the chili pepper.

## Introduction

A long-standing question in biology is how genetic variation contributes to phenotypic variation. The extraordinary diversity observed in plant phenotypes is predominantly due to differences in gene regulation, rather than major changes in the products of the genes themselves (Cubillos et al. 2012; Meyer and Purugganan 2013). Gene expression differences result from mutations both in local noncoding regulatory DNA sequences that act in *cis*, such as enhancers and promoters, and in regulatory components that act in *trans*, such as transcription factors (TF) and cofactors (Wittkopp and Kalay 2012). *Cis-* and *trans*-regulatory elements underlie complex phenotypic traits, including the dramatic changes in fruit size and shape typical of plant domestication (Doebley et al. 2006). It is likely that genome-wide *cis-* and *trans*-regulatory elements interact in gene regulatory networks, and behave in an “omnigenic” pattern in which there are peripheral genes that interact with a core of pleiotropic *trans*-acting genes, and even small changes in their expression can be magnified to produce a dramatic phenotype impact (Liu et al. 2019).

Plant domestication is an ideal scenario to explore the relative contribution of *cis-* and *trans-*regulatory variations in the generation of novel phenotypes. Domestication is an ongoing process that was initiated ∼12,000 years ago, in which often dramatic morphological divergences from the wild ancestors arises due to artificial selection of desired crop phenotypes (Zeder 2015). Moreover, even as hundreds of generations pass by in the course of plant domestication (Gepts 2004), it is still much faster than morphological changes during natural evolutionary processes such as speciation events, that take place in the order of millions of years and millions of generations (Beddows et al. 2017).

The genetic architecture of plant domestication phenotypes described by quantitative trait loci (QTL) mapping and molecular complementation analyses show that mutations affecting regulatory, rather than protein coding regions, are indeed the major source of variation associated with morphological change; such as increased fruit size in tomato (Cong and Tanksley 2006) and shoot architecture in maize (Wang et al. 1999). Furthermore, gene expression differences have been correlated with domestication in well-known crops such as tomato and maize (Springer et al. 2019).

Allele-specific expression (ASE) analysis aims to characterize genome-wide regulatory effects by quantifying the relative contribution of paternal alleles to the transcriptome of an F_1_ hybrid, which, in combination with data obtained from the parents individually, allows the assessment of *cis-* and *trans-*regulatory variations. ASE analyses in barley (Haas et al. 2019), maize (Lemmon et al. 2014; Aguilar-Rangel et al. 2017), tomato (Albert et al. 2018), and pearl millet (Rhoné et al. 2017) have highlighted the relevance of regulatory variants in plant domestication. Complementary studies to map expression-QTL linked to variation in transcript abundance have identified key *cis*-regulatory variants associated with variation in crop morphology that impact the expression of thousands of genes in *trans* (Ranjan et al. 2016; Wang et al. 2018). Moreover, recent evidence in maize shows that its domestication genes are interconnected by TFs (Studer et al. 2017). Together this evidence suggests that it is the sum of the interactions of *cis-* and *trans* effects that gives rise to the regulatory landscape of domesticated plant morphology.

Chili pepper is a crop of neotropical origin, domesticated in Mexico (<6,000 years ago) (Perry et al. 2007) with cultural and economic importance worldwide (http://www.fao.org/faostat/; last accessed November 15, 2019). It is appreciated for its broad range of phenotypic variation in fruit shape, size, color, and pungency. Therefore, the evolution of fruit shape, size, and maturation during domestication of chili pepper is of key biological and economic interest. In its center of domestication alone, there are ∼40 phenotypically different varieties closely associated to various sources of traditional and modern knowledge, and there are hundreds of varieties developed and used worldwide (Hill et al. 2013). In this study, we evaluated the contribution of regulatory variation to gene expression divergence between a cultivated chili pepper, *Capsicum annuum annuum*, “puya,” with a shape that is representative of most cultivated Mesoamerican peppers, and its wild relative *Capsicum annuum glabriusculum*, to explore the regulatory mechanisms, inheritance, and coexpression patterns of genes associated with fruit morphology. Our combined use of ASE and network analyses reveals that the domesticated chili pepper fruit is a result of recessive patterns of expression in downregulated genes associated with fruit and embryo development. and that, few *cis*-regulated genes that affect many *trans*-acting genes provide a genome-wide mechanism for the rapid morphological changes expected during the process of its domestication.

## Results

### Morphometric and Gene Expression Analyses of the Fruits of Wild and Cultivated Chili and Their F_1_ Hybrid Reveal Partially Recessive Inheritance of Cultivated Characters

**Fig. 1.**
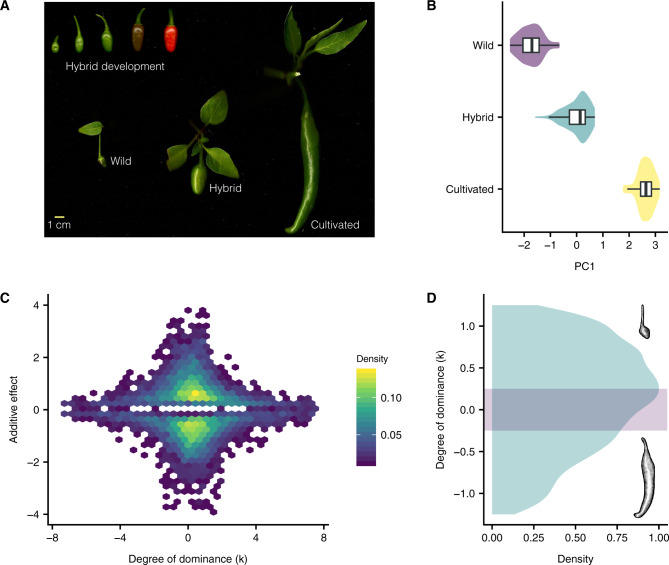
Comparative morphometrics and gene expression analysis reveals partial recessiveness of the cultivated phenotypes. (*A*) Development of the F_1_ hybrid fruit at 10, 20, 40, and 60 DAA (top); and the three genotypes at 40 DAA. (*B*) Plots showing the overlap of PC1 between the wild and hybrid genotypes. Boxplots depict the median and quartile values. (*C*) Hexabin density plot showing the relationship between the additive effect (*a*) and the degree of dominance (*k*) for 8,521 DEGs between parents. Small additive effects result in larger estimates of *k*. Hexagons are bins of genes and are color-coded according to the normalized frequency (density) of each bin. (*D*) The distribution of *k* is plotted within the nontransgressive inheritance interval. The purple bar depicts the additivity interval (−0.25, 0.25).

To explore the genetic mechanisms underlying the change in fruit phenotypes as a result of chili pepper domestication, we evaluated morphology and gene expression in the cultivated (C) accession “puya” (*Capsicum annuum* var*. annuum*), the wild (W) ancestor “chiltepín” (*Capsicum annuum* var. *glabriusculum*), and their F_1_ hybrid (C×W). At harvest (40 days after anthesis; DAA), fruit size and shape in C×W was more similar to W than C parent (fig. 1*A*). In a principal component (PC) analysis, PC1 accounted for 97% of the total variation in fruit morphology characteristics and separated C from a group containing W and C×W (fig. 1*B* andsupplementary fig. S1*A*, Supplementary Material online). Fruit size and shape of cultivated chilis has been reported previously to be recessive to that of wild varieties (Kaiser 1935). We evaluated dominance at the level of total transcript abundance with respect to each individual gene detected in our transcriptomes and found a bias toward the W parental level in C×W. Overall, we found 8,521 (50.3% of total) differentially expressed genes (DEGs) between chili pepper parents, a value lower than that observed between wild and cultivated maize (70%) (Lemmon et al. 2014) or between small- and big-fruited tomatoes (81%) (Koenig et al. 2013; Albert et al. 2018), but higher than between wild and cultivated bean (1%) (Bellucci et al. 2014), between two African *Coffea* subspecies (33%) (Combes et al. 2015), and between small and large pumpkins (40%) (Xanthopoulou et al. 2017). Although we found a similar number of genes to be up- or downregulated in C with respect to W (4,123 showed upregulation in C, whereas 4,398 were downregulated [binomial test, *P *=* *0.003], slight deviating from equal). An analysis of functional categories found that the set of genes downregulated in C was enriched for genes associated with fruit and embryo development (supplementary fig. S2*A*, Supplementary Material online). Looking more closely at the inheritance of transcript levels in C×W, we estimated dominance (*k*) on a scale from −1.25 (C completely dominant) to 1.25 (C completely recessive), where *k *=* *0 indicated additivity, and any value beyond |1.25| was considered transgressive, which means the hybrid has either greater or lower values than either parent (fig. 1*C*) (also see Materials and Methods). Genes for which *k* was near zero were found to display a greater magnitude of differential expression between C and W parents. Conversely, genes for which *k*>|2| tended to show smaller differences between the parents. The majority of genes fell within the range of *k*<|1.25|, with more instances of the C pattern being partially recessive (positive values of *k*. fig. 1*D*). Recessive patterns of expression were most common in downregulated genes in C, a group enriched for functional categories associated with fruit and embryo development, including embryo development (GO: 0009790), reproductive structure development (GO: 0048608), and seed (GO: 0048316) and fruit development (GO: 0010154; supplementary fig. S2*B*, Supplementary Material online). In contrast, the expression of genes associated with abiotic stress, specifically heat stress, was transgressive in C×W, whereas expression of genes categorized in basic cellular functions were largely additive (supplementary fig. S2*B*, Supplementary Material online).

### *Trans*-Acting Variants Have the Largest Effects on Gene Expression Differences between the Cultivated and the Wild Chili Pepper Fruit

To estimate the contribution of the two parental alleles to the levels of expression observed in C×W and to investigate the contribution of *cis-* and *trans*-regulatory variations, we performed an ASE analysis on 5,077 transcripts associated with informative single-nucleotide polymorphisms (SNPs). Transcripts with *cis*-regulatory changes were identified as allelic imbalances in C×W transcriptomes, whereas transcripts with *trans*-regulatory changes were detected by comparing allele-specific differences in C×W to the degree of differential expression between the C and W parents. We found that *trans*-acting variation has the largest effect on both the proportion and the magnitude of gene expression differences (testing against the neutral expectation of an equal proportions of *cis* and *trans. z *=* *26.29; *P *<* *0.001). Out of 5,077 transcripts with detectable ASE, 31% (1,007) showed differences in expression in F_0_ or F_1_ generations. From this set ∼95% (960) transcripts showed significant *trans*-regulatory variation, ∼10% (102) transcripts showed significant evidence of *cis*-regulatory variation, and ∼5% (55) of these transcripts showing evidence of both regulatory mechanisms.

**Fig. 2.**
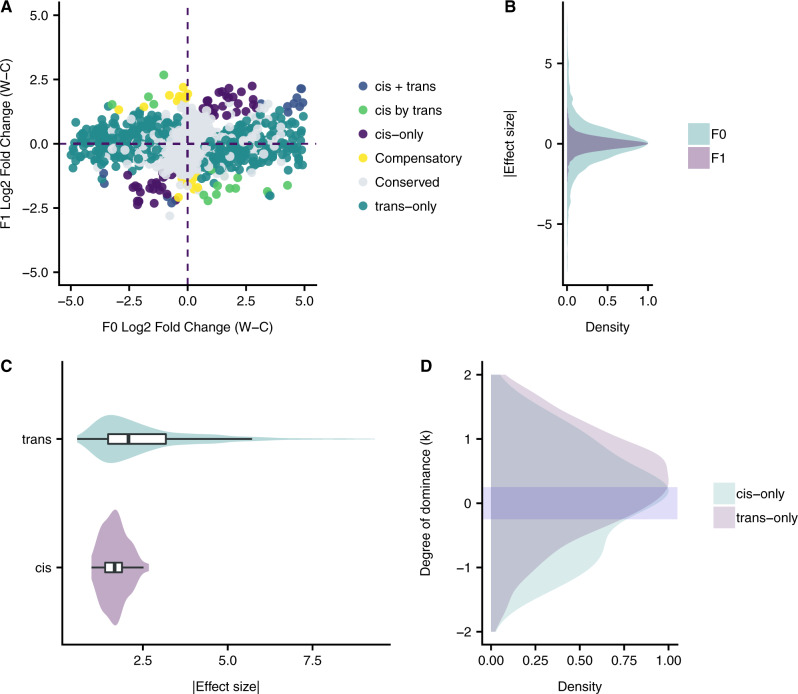
Dissection of *cis-* and *trans-*regulatory changes associated to chili pepper fruit domestication, showing there is greater number of genes with *trans*-acting variation. (*A*) The log_2_-fold change of the wild (W) versus the cultivated (C) allele in the F_0_ and F_1_ generations is plotted for the 5,077 transcripts employed in the ASE analysis. Each dot represents a single transcript and is color-coded according to the category of regulatory variation. (*B*) The distribution of the effect sizes of expression differences for the wild and cultivated alleles for both the F_0_ and the F_1_ generations illustrating a wider range for F_0_. (*C*) Distribution of the effect sizes (log_2_-fold change) for genes with *cis-* and *trans-*regulatory changes shows that *trans* effects (median = |2.1|) are larger than *cis* (median = |1.75|) effects. (*D*) The distribution of the degree of dominance (*k*) is plotted for both genes with *cis*-only and *trans*-only within a |2| interval. The purple bar depicts the additivity interval (−0.25, 0.25).

Based on the categories proposed by McManus et al. (2010) (see Materials and Methods), we identified 47 transcripts as regulated in *cis*-only and 905 as regulated in *trans*-only (fig. 2*A*). A further 19 transcripts displayed *cis-* and *trans* variations acting in the same direction (*cis* + *trans*), and 20 showed regulation by both *cis* and *trans*, but in opposing directions (*cis* by *trans*) (fig. 2*A*). Finally, 16 transcripts showed *cis-* and *trans* variations, but equal expression in the C and W parents, suggesting that *cis-* and *trans* variations compensate each other. The *trans*-only gene set is enriched in functional categories related to fruit morphology (GO: 0010154); seed development (GO: 0048316); and embryo development ending in seed dormancy (GO: 0009793) (supplementary table S3, Supplementary Material online), in accordance with the pattern seen in the C downregulated (supplementary fig. S2*A*, Supplementary Material online) and partially recessive gene sets, respectively (supplementary fig. S2*B*, Supplementary Material online). Although no directional bias in allelic expression differences were found either between the parents on in the hybrid (fig. 2*B*), the absolute effect sizes of *trans*-regulatory expression divergence between C and W were greater than ASE effects seen in C×W (*trans*-median = 2.125; ASE median = 1.75; Wilcoxon’s rank-sum test; *P* < 0.001; fig. 2*C*), similar to the pattern reported in switch grass (Lovell et al. 2016). In addition, expression differences associated with *trans*-regulatory variation showed a greater range than those associated with *cis*-regulatory variation (fig. 2*C*). For genes, for which both *cis*- and *trans*-acting variations acted in the same direction, the effect size was larger than for genes for which *cis*- and *trans*-variations acted in opposite directions (supplementary fig. S3*A*, Supplementary Material online). Thus, across the gene set, as the magnitude of differential expression between the parents increases, so does the importance of *trans*-regulatory variation (supplementary fig. S3*B*, Supplementary Material online). Finally, we confirmed the expectation that nonadditive inheritance of gene expression levels in C×W (assessed at the level of absolute expression) was associated with *trans*-regulatory variation (assessed at the level of allele-specific contribution; fig. 2*D*).

### Genes with *Cis*-Regulatory Divergence and Their *Trans*-Acting Interactions Are Associated with Fruit Development and Maturation

**Fig. 3.**
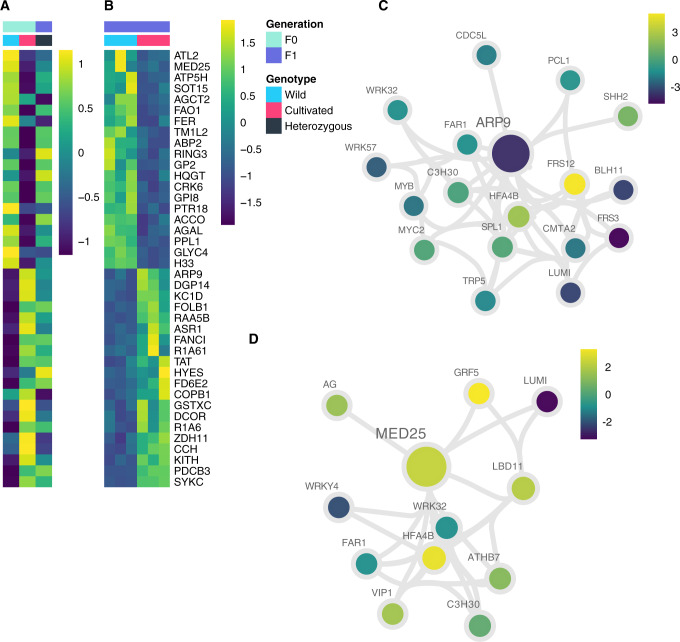
Heatmaps of genes with *cis*-only regulatory divergence and coexpression networks for a set of genes with significant *cis*-only (FDR<5%) reveals two clusters of genes as down- or upregulated. (*A* and *B*) Heatmaps displaying the expression level based on normalized read counts as a *Z*-score for each gene enlisted using their Uniprot Identifiers (rows). The A panel shows total expression for each genotype (W, C, and C×W) as the *Z*-score of the median among the three libraries. The inheritance of each gene is depicted by their colors in the parents and the F_1_ hybrid. The B panel depicts the *Z*-score of the allele-specific expression for the three libraries in C×W. (*C* and *D*) AracNe-based coexpression networks of *ARP9* and *MED25 cis*-only genes based on tomato orthologs. Each network shows the closest neighbors of the selected node. Nodes are color-coded according to the log2FC(W/C) and node sizes correspond to betweenness centrality, which is a measure of their connectivity and potential pleiotropy. The length of the edges is based on the values of the mutual-information value, which reflects the level of coexpression between them (shorter edges mean higher coexpression between nodes).

Although the largest gene expression differences between W and C were the result of *trans*-regulation, selection during domestication must ultimately act on the upstream *cis*-variants that drive these *trans* effects. A total of 102 genes showed *cis*-regulatory differences dispersed broadly across all 12 chromosomes of chili pepper. They were mostly located far from the centromeres and in the chromosome arms, colocalizing with gene-rich regions (supplementary fig. S4, Supplementary Material online). As would be expected, the majority of *cis*-regulated genes displayed additive inheritance (fig. 2*D*), as has been observed in fruit fly, maize, and yeast hybrids (Metzger et al. 2017; McManus et al. 2010; Lemmon et al. 2014). About 25% of these genes were associated with fruit development and maturation processes. *Cis*-regulated genes associated with ripening are downregulated in the cultivated plant (fig. 3*A* and *B*), suggesting loss-of-function associated with delayed maturation in domesticated chili. These genes include *RING3*, which is involved with protein degradation during the maturation of the fruit (Stone and Callis 2007), *GP2* and *AGAL*, involved in the remodeling of the cell wall (Rao and Paran 2003; Tsaniklidis et al. 2016). AGAL plays a role in carbohydrate degradation, possibly related to changes in organoleptic properties, giving the sweet taste of the fruit, a trait unique to the cultivated chili pepper. Of the few upregulated genes, *DCOR* is involved in alteration of the expression pattern of ripening specific genes (Pandey et al. 2015) and is also upregulated in the presence of pathogens (virus and bacteria) (Yoo et al. 2004), and *ASR1* is active in delaying maturation (Jia et al. 2016).

Two *cis*-genes were potentially associated with greater fruit size in the cultivated parent, *ARP9* and *MED25*. *ARP9* encodes a nuclear actin-related protein that orchestrates developmental transitions by remodeling chromatin complexes via its heterodimeric obligate interaction with *ARP7* in the RSC (remodels the structure of chromatin) complex (Szerlong et al. 2003). The function of *ARP9* in plants is unclear, however, the loss-of-function mutant *arp7* in *Arabidopsis thaliana* shows defects in cellular and macroscopic features product of deregulation of cell proliferation and expansion, resulting in an overall organ size decreasing. (Kandasamy et al. 2003; Meagher et al. 2007). Therefore, it is possible that in cultivated chili pepper, the increase in fruit size is associated with the gain of function of *ARP9* via *cis*-regulatory variation.

*MED25* encodes a member of the Mediator multiprotein transcriptional complex and has been associated with jasmonate signaling, defense responses, and negative regulation of cell proliferation and expansion. The loss-of-function mutant of *MED25* resulted in larger organs in *A. thaliana* (Xu and Li 2011; Wang et al. 2019), thus, it would be possible that its downregulation in cultivated chili peppers is linked to larger fruit size. Additionally, among our *cis*-only candidates, we observed transcripts of an ortholog of the *Arabidopsis FANCM* crossover suppressor (Crismani et al. 2012), to be more abundant in C than W (fig. 3*A* and *B*), although its direct association with the domestication syndrome remains unexplored. Interestingly, five of our *cis*-only genes are found within genomic regions previously identified with signals of positive selection (Qin et al. 2014) (supplementary fig. S4, Supplementary Material online). Congruently, these regions contain genes annotated as fruit growth and ripening regulators.

To investigate the capacity for the *cis*-regulatory variants to drive the observed *trans* effects, we projected chili orthologs onto a tomato coexpression network containing 7,830 genes and ∼97,000 interactions. We then extracted individual subnetworks containing selected *cis*-regulated candidates (supplementary fig. S5, Supplementary Material online). The coexpression network of *ARP9* was enriched in TF genes (*P* < 0.001), consistent with a role as an upstream *trans-*regulator of many other genes in the hierarchy of the network (fig. 3*C*). A similar pattern has been observed in maize (Lemmon et al. 2014; Wang et al. 2018), in which genes regulated in *cis*, in the same sense of our *cis*-regulatory hubs, are often the *trans*-acting regulators. There were a number of light-sensitive genes, including *FRS3* and *FRS12* (Ma and Li 2018) downstream of *ARP9*, potentially contributing to regulation of fruit development. *FRS12*, which is downregulated in the cultivated chili pepper, is 1 of 12 TFs of the *FAR1* family, and acts downstream of phytochrome A to regulate photomorphogenic development and coordinate the activation of circadian clock evening components (Ritter et al. 2017). It is interacting closely in our network with slightly downregulated in the cultivated *PCL1*, a *MYB* TF known as LUX that is involved in circadian clock oscillations (Hazen et al. 2005). Loss-of-function of *FRS12* in *A. thaliana* results in early flowering plants with overly elongated hypocotyls mainly in short days, although it may be involved in other functions in the chili pepper fruit (Ritter et al. 2017). The *MED25* network contained a number of TFs associated with fruit growth, fruit ripening, and stress responses (fig. 3*D*). MED25 and MYC2 are corecruited to the promoter of target genes of MYC2, and as a complex, they regulate jasmonate-mediated signaling (Wang et al. 2019). In our coexpression networks, *MYC2* does not belong to the closest neighbors of *MED25* but they interact in the general network by means of *HFA4B*, a transcriptional regulator of heat shock proteins (supplementary fig. S5, Supplementary Material online), which is also highly coexpressed with a few WRKY TFs in our networks. The *MED25* network also includes the downregulated *AG* in the cultivated parent, one of the master regulators of floral and late fruit development. Downregulation of *AG* in tomato resulted in decreased levels of expression in genes associated with fruit ripening and cell wall metabolism, possibly modulating fruit softening, aroma, and shelf life (Itkin et al. 2009).

## Discussion

The morphology and transcriptome of the fruit of the wild×domesticated chili hybrid revealed a recessive pattern of inheritance in the domesticated chili pepper. The downregulation and the recessiveness of genes involved in fruit ripening in cultivated chili suggest that artificial selection during its domestication process acted on loss-of-function variants. Although such variants might be deleterious in the wild, they provide a ready source of variation to generate phenotypes desirable under domestication (Pickersgill 2007; Huber et al. 2018). In cultivated tomato, pumpkin, and pear, increased fruit size results from the reduced activity of repressors of cell proliferation or enlargement (Koenig et al. 2013; Xanthopoulou et al. 2017; Li et al. 2019). QTL mapping studies have identified orthologous and syntenic loci controlling fruit size in both tomato and chili pepper (Paran and van der Knaap 2007), suggesting genetic and molecular parallelism during the domestication of these two closely related neotropical species, although this hypothesis still needs to be tested explicitly.

Our results support a growing collection of studies that implicate *cis*-regulatory variation in phenotypic evolution (Wittkopp and Kalay 2012) and highlight the role of *trans*-regulatory variation—with the potential to impact multiple downstream targets—as an attractive mechanism of rapid phenotypic change. The majority of our DEGs were associated with *trans*-acting variation. The predominance of *trans* divergence in the domestication of the chili pepper is also congruent with observations of regulatory patterns in other organisms with short times of divergence (<10,000 years) (Metzger et al. 2017; Yvert et al. 2003), well within the time scale of domestication processes.

A pair of transcriptional regulators/coactivators, *ARP9* and *MED25*, associated with organ size variation, are good domestication candidates. Variation in the regulatory network of *ARP9* could contribute to enlargement in the fruits of cultivated chili pepper, mostly as a *trans-*acting regulator, with light-sensitive genes downstream of ARP9 that contribute to the chili pepper fruit phenotype. Likewise, *MED25* coexpresses with TFs that are involved in fruit growth, fruit ripening, and stress responses which could be coregulated upstream of these transcriptional networks.

The transition of the morphology of the wild chili pepper to the current cultivated fruit likely involved recruitment of a few key *cis*-acting mutations that had pleiotropic effects, in an “omnigenic” fashion, rather than single genes acting independently to determine the trait (Liu et al. 2019). This would be similar to what has been found in maize in which *cis*-acting mutations impact the structure of regulatory networks via pleiotropic *trans*-acting variation (Studer et al. 2017; Stitzer and Ross-Ibarra 2018). This scenario is plausible for chili pepper given the short amount of evolutionary time in which its domestication has occurred (<6,000 years ago), the polygenic nature of the fruit morphology, and the astounding fruit variation currently observed in pepper varieties. It remains to be seen if the contributions from *trans*-regulatory variants with large effect size are correlated to factors such as the divergence times between parents among other domesticated chili pepper varieties and species, tomato or other domesticated members of the Solanaceae. Another remaining question is if *cis*-acting variation detected in this study is pervasive among domesticated chili peppers, or if each accession has alternative subnetwork structures of different *cis*-only regulated genes that would retain similar functions controlling the domesticated fruit morphology. This would shed light on whether the chili pepper fruit domestication process is a result of convergence in regulatory mutations affecting the same few pleiotropic genes with *cis-*only variation, or if there are many ways to regulate the transcriptome leading to the domesticated phenotype.

The identification of a common, shared panel of *cis*-regulatory variants and their *trans*-regulatory targets for crop improvement would be of interest for agriculture, especially if those variants are linked to variation in the increasingly endangered wild relatives. Well-characterized *cis*-regulatory regions in the wild relative that are hotspot eQTL of key gene networks, are ideal candidates for crop improvement using genome editing, genomics assisted breeding, and genome rewilding, which focuses on the recovery of genetic variation of value from wild relatives (Swinnen et al. 2016). In the light of major habitat and climatic changes, the discovery of rapid mechanisms of phenotypic change derived from regulatory variation in crop wild relatives and existing crops themselves is of great value.

## Materials and Methods

### Plant Material and Growth

Fruit of the cultivated (C) variety “Puya” *Capsicum annuum* var*. annuum* was obtained from Irapuato, Guanajuato, Mexico (20.67°N, 101.35°W). Fruit of the wild (W) pepper “Chiltepín” *Capsicum annuum* var*. glabriusculum* was collected from a wild population located in El Patol, Querétaro, Mexico (20.79°N, 99.87°W). Seeds were extracted from dried fruits, washed with a 10% v/v bleach solution, scarified with 0.05 N HCl at 35 °C by 30 min, and rinsed with distilled water prior to planting in 0.5-l pots containing a 3:1:1 mix of peat moss, vermiculite.

Plants were grown in a greenhouse in Irapuato, with temperature controlled in the range 18–28 °C, and natural light and humidity. Plants were fertilized every 2 weeks with standard NPK fertilizer. Once plants reached sexual maturity, F_1_ hybrids were produced using C as female and W as male, as previously described (Pickersgill 1971). Additional flowers were left to self-fertilize to generate C and W parental stocks.

### Analysis of Fruit Morphology

At 40 DAA, 20 fruits per plant were collected from each of 10 C×W, 5 C, and 5 W plants, and scanned at 300 dpi using a standard flatbed scanner. Images were analyzed using the ImageJ software package (Rueden et al. 2017) to extract the following shape descriptors: area (A), minor axis (MiA), major axis (MaA), aspect ratio (AR = MaA/MiA), roundness (Ro = 4×[(A)/π(MaA)^2^]), and circularity (Ci = 4π[(AR)/(perimeter)^2^]). The morphometric data were log10 or arsine transformed and subjected to a principal component analysis (PCA) to obtain the main trends of variation (PCs). Individual traits (area, aspect ratio, circularity, major, and minor axes) were highly correlated with PC1 (supplementary fig. S1*A*, Supplementary Material online), so we decided to use PC1 as a trait in itself to describe the parental and the hybrid morphology, and also because PCs can capture complex phenotypes well (Rosas et al. 2013). The individual contributions of area, major axis, minor axis (size descriptors), as well as aspect ratio and circularity (shape descriptors) to the PC1 were 0.74, 0.52, 0.22, 0.30, and 0.13, respectively (supplementary fig. S1*A*, Supplementary Material online). The distribution of the five main traits are shown individually for each genotype in supplementary figure S1*B–F*, Supplementary Material online. Knowledge of the trait inheritance is an important component of quantitative genetic approaches, as it determines the response of the trait to selection and the nature of genetic changes driving phenotypic divergence. We were interested in the continuous distribution of inheritance in addition to discrete categories, which we consider to be subjective. As we see it, a distribution provides a less-biased way to explore how allelic variants from wild and cultivated parents interact in the heterozygous hybrid, or their mode of gene action. It also opens the possibility of distinguishing bins or categories of incomplete dominance, which are often ignored in studies of inheritance of gene expression. To estimate dominance, we used a measure of the degree of dominance (*k *=* d*/*a*), that estimates the relationship between the additive and dominance effect. We used *k* to evaluate gene action associated with variation between C and W and gene expression, measured as transcript abundance. The additive effect (*a*) provides a measure of the degree of change in the phenotype that occurs in the hybrid with the substitution of one wild allele for one cultivated allele, or vice versa. The dominance effect (*d*) measures how the phenotype in the F_1_ hybrid deviates from a midpoint of the two homozygous classes, in this case each of the parents. We estimated the additive (*a* = (W−C)/2) and dominant (*d* = C×W−[(W+C)/2]) effects and calculate the degree of dominance (*k *=* d*/*a*) of all morphometric traits. Both PCA and *k* estimates were carried out in R (v. 3.5.2, CRAN).

### RNA Extraction, Library Preparation, and Sequencing

Total RNA was extracted from the placenta and pericarp tissue of fruits at 40 DAA, for a single fruit harvested from each of three individuals for each genotype (C, W, or C×W), for a total of nine samples. Prior to collection of placenta and pericarp tissue, fruits were quickly cleaned with ethanol, and dissected to remove the seeds. RNA was extracted using TRIzol (Invitrogen) following the manufacturer’s recommendations, with the following modifications: TRIzol reagent was incubated at 56 °C prior to use; an additional 24:1 chloroform–isoamyl alcohol extraction was performed; RNA precipitation was done using one volume of saline solution (0.8 M sodium citrate + 1.2 M sodium chloride) for one volume of isopropanol; an additional 70% ethanol wash was performed. Total RNA concentration was quantified using a NanoDrop 2000 spectrophotometer (Thermo Scientific Nanodrop), and RNA integrity was evaluated by electrophoresis on a 1.5% nondenaturing agarose gel. For sequencing, cDNA libraries were prepared from 3 μg of total RNA (RIN ≥ 5) using the Illumina TrueSeq RNA Library Preparation Kit v3. The resulting nine cDNA libraries were sequenced on a half-lane of the Illumina Hi-Seq 4000 platform, yielding ∼350,000,000 million 2×101-bp paired-end reads.

### Differential Expression between Parents and Assessment of Mode of Gene Action

Sequencing reads were first filtered using Trimmomatic v. 0.32 (Bolger et al. 2014) to remove adapters and discard reads with a per-base quality <25 or a total length <70 base pairs. Trimmed reads were then analyzed with the FastQC software (https://www.bioinformatics.babraham.ac.uk/projects/fastqc/; last accessed October 20, 2019) to verify their quality, length distribution, and the absence of Illumina adapters. Expression profiles of absolute total transcript abundance were generated by pseudoaligning the sequencing reads to a chili pepper (CM334 v.1.55) reference transcriptome (Kim et al. 2014) using Kallisto ver 0.43.0 (Bray et al. 2016) in pair-end mode with parameters -b 100, -t 16, and –bias. About 71–86% of the reads belonging to the cultivated libraries mapped to a single feature in the reference transcriptome, whereas the percentage of mapped reads for the W and F_1_ libraries ranged between 66–73% and 61–73%, respectively (supplementary table S1, Supplementary Material online). Transcripts with a per-genotype median of read counts smaller than five were excluded for further analysis. Transcript abundance estimates were used to test for differential expression (FDR 5%) using the DESeq2 R package (Love et al. 2014) which measures differential expression by fitting a generalized linear model (GLM) following a negative binomial distribution, and scaling library size by normalizing factors. After normalizing read counts, expression profiles for 16,938 gene models were generated, comprising 48.5% of the total gene models. We assessed transcriptional divergence between cultivated and wild transcriptomes by fitting a GLM to read count data using a likelihood ratio test (LRT) (FDR = 5%) via DESeq2. The degree of dominance index (*k*) was employed as a proxy to investigate the mode of gene action (additive, dominant-recessive, transgressive) of each transcript. Normalized transcript abundance was collapsed to a per-genotype median value and *k* was estimated in a row-wise fashion via a custom R script.

### Generation of ASE Data

To identify and quantify the parental-specific reads in the F_1_ hybrid transcriptomes, a pseudoreference approach was employed. Briefly, reads of the two sets of parental libraries were mapped to the CM334 v.1.55 reference genome (Kim et al. 2014) with STAR (Dobin et al. 2013). Resulting BAM files were then used to call SNPs via the GATK toolkit (Poplin et al. 2017) using the Haplotype Caller mode with default parameters. SNPs with homozygous genotype for the alternative allele and a read depth of at least 4× were retained and used to produce a pseudoreference for each parent via the FastaAlternateReferenceMaker function of the GATK tool kit. C×W libraries were independently mapped to each of the two pseudoreferences via STAR and then analyzed by GATK as mentioned earlier. The read mapping of the three F_1_ libraries against the cultivated pseudoreference (F_1_–C) retrieved 54,910, 39,401, and 59,493 SNPs, respectively, whereas the alignment against the three wild pseudoreference (F_1_–W) yielded 34,237, 24,134, and 36,394 SNPs. These SNP sets were merged and only the shared positions among the six SNP sets were retained, then assigned to gene models and filtered by the quality of the alignment and depth of coverage (QUAL>30; DP>20). In order to capture SNP sites with unambiguous information regarding parental-specific expression, only SNPs at biallelic positions with heterozygous and reciprocal genotypes between F_1_–C and F_1_–W alignments (e.g., A/G; G/A) were retained (supplementary table S2, Supplementary Material online). To create the ASE profiles, the read depth of each allele was retrieved from the VCF files only if the values of per allele read depth showed reciprocity between C×W–W and C×W–C. Finally, the per-allele read depth was summed over SNPs mapping to the same gene model, resulting in ASE profiles for 5,077 transcripts across three replicates.

### Modeling *Cis-* and *Trans-*Regulatory Divergences via GLM

At the transcriptional level, gene expression is governed by interactions between *cis-* and *trans*-regulatory elements, thus, mutations affecting each of them or both, impact the expression of downstream genes. The role of *cis-* and *trans-*regulatory variations on transcript abundance differences between parental two accessions can be inferred by assessing the allelic imbalance of two alleles in a common *trans* environment in a F1 hybrid. In this scenario, transcripts with allelic imbalance suggest that linked *cis*-acting variation is responsible for expression differences between parents.

Using the DESeq2 R package, we modeled ASE as the differential expression between the two alleles in C×W transcriptomes. A GLM was fitted and an LRT was run to test two models: reduced = ∼ 1; full = ∼ allele. To test for *trans*-acting variation, we fitted a GLM to test whether at least one parental allele was differentially expressed between the F_0_ and F_1_ generations. We tested two models with an LRT: reduced = ∼ allele + generation; full = ∼ allele + generation + allele×generation. Then, a custom R script was written to sort transcripts into seven categories of regulatory divergence according to McManus et al. (2010). From this set, a total of 2,339 (69%) transcripts analyzed showed conserved expression in both F_0_ and F_1_ and across generations, whereas 1,517 transcripts were classified as ambiguous because the observed patterns of significance have no biological interpretation at the transcriptional level (McManus et al. 2010). *Cis*-only was defined as significant differential expression between C and W in F_0_, evidence of *cis* divergence with no evidence of *trans* effects. *Trans*-only were defined as significant differential expression between C and W in F_0_, evidence of *trans* with no evidence of *cis* divergence. We focused on the transcripts with nonconserved or nonambiguous expression. We estimated the relative contribution of *cis-* and *trans*-regulatory changes in producing expression differences in F_0_ by binning genes according to their effect sizes (|parentLog2FC(W/C)|), and by computing the *cis* percentage (|*cis*|/(|*cis*|+|*trans*|)).

### Gene Ontology and Coexpression Analyses

Gene ontology (GO) term enrichment analyses were performed on the different gene sets using the topGO R package (Alexa et al. 2006). CM334 gene models were blasted (Altschul et al. 1990) against the Swiss-Prot database (Bateman 2019) and GO terms retrieved from the UniProt database by importing a matrix with UnProtKB IDs (http://www.uniprot.org/uploadlists/; last accessed August 10, 2019) for each annotated gene model. A reciprocal best hit approach was employed to identify orthologous transcripts between CM334 and tomato via BLAST. Then, 80 RNA-seq-based RPKM-normalized expression profiles from placenta and pericarp were retrieved from the tomato expression atlas database (http://tea.solgenomics.net/; last accessed May 13, 2019) and used as input to ARACNe-AP (Lachmann et al. 2016) to infer mutual information among TFs and non-TF genes. The coexpression matrix was imported into Cytoscape ver 3.7.1 (Shannon 2003) to visualize the graph and perform network analyses. We refer to upstream as the assumed position of the gene in the hierarchy of the network, based on the betweenness centrality measurements (Peter and Davidson 2017). The precise gene model variants that would result in differences in gene expression of these genes, remain to be identified. Heatmaps and circular plots were plotted using the pheatmap (Kolde 2019) and shinnyCircos (Yu et al. 2018) R packages, respectively. 

## Supplementary Material

msaa027_Supplementary_Data

## Notes

A.C.J. and R.H.S. designed the project. E.D.-V. performed the research and analyzed the data. A.C.J. and E.D.-V. wrote the article. R.H.S. critically revised the article. A.C.J. funded the project.
