# Supplementary material for: Cis- and Trans-Regulatory Variations in the Domestication of the Chili Pepper Fruit
Source: Mol Biol Evol. 2020 Feb 7;37(6):1593–603. doi: 10.1093/molbev/msaa027 (PMC7253206; doi:10.1093/molbev/msaa027)
Supplement: msaa027_Supplementary_Data [file msaa027_supplementary_data.pdf]

Supplementary Tables

Table S1. Overview of transcriptome analysis via Illumina Hi-seq 4000 sequencing

	Cultivated Parent			F1 Hybrid			Wild Parent		
	C_1	C_2	C_3	CxW_1	CxW_2	CxW_3	W_1	W_2	W_3
Total PE reads (millions)[a]	30.24	28.93	33.63	24.17	24.45	29.60	30.87	24.55	27.43
Filtered reads (millions)	14.13	11.43	15.68	13.11	11.15	11.48	14.13	11.43	12.70
Mapped reads (millions)	10.04	9.82	11.91	9.52	6.76	8.33	9.90	7.62	9.26
Number of expressed transcripts (percentage)[b]			20,456 (58.61%)			20,053 (57.46%)			19,702 (56.45%)

[a]Parental and hybrid results are total number of raw pair-end (PE) reads, regardless of allele specificity.
[b]Transcripts with at least 5 read counts in at least 47% of the samples. Percentage values reflect the portion of the whole transcriptome expressed in each RNA-seq library, considering 34,899 predicted transcripts.

Table S2. Details about the Allele-Specific SNPs in F1 hybrid libraries.

	Vs Cultivated Pseudo-reference			Vs Wild Pseudo-reference		
	CxW_1	CxW_1	CxW_1	CxW_1	CxW_1	CxW_1
Number of raw heterozygous SNPs[a]	54,910	39,401	59,494	34,237	24,134	36,394
Shared SNPs between libraries			17,138			29,079
SNPs with reciprocal genotypes[b]						10,404
Number of transcripts bearing parental-specific alleles						5,077
Number of diagnostic SNPs per transcript (mean)						2.03

[a]Filtered SNPs with at least 10 reads supporting the polymorphism

[b]Heterozygous SNPs that retrieve reciprocal genotypes when F1 hybrid reads are mapped against each of the two pseudo-references.

Table S3. Gene ontology terms with significant (p <= 0.1) enrichment for the trans-only gene set.

GO ID	Description
GO:0003006	developmental process involved in reproduction
GO:0007275	multicellular organism development
GO:0009790	embryo development
GO:0009791	post-embryonic development
GO:0009793	embryo development ending in seed dormancy
GO:0010154	fruit development
GO:0022414	reproductive process
GO:0032501	multicellular organismal process
GO:0032502	developmental process
GO:0048316	seed development
GO:0048608	reproductive structure development

Supplementary Figures

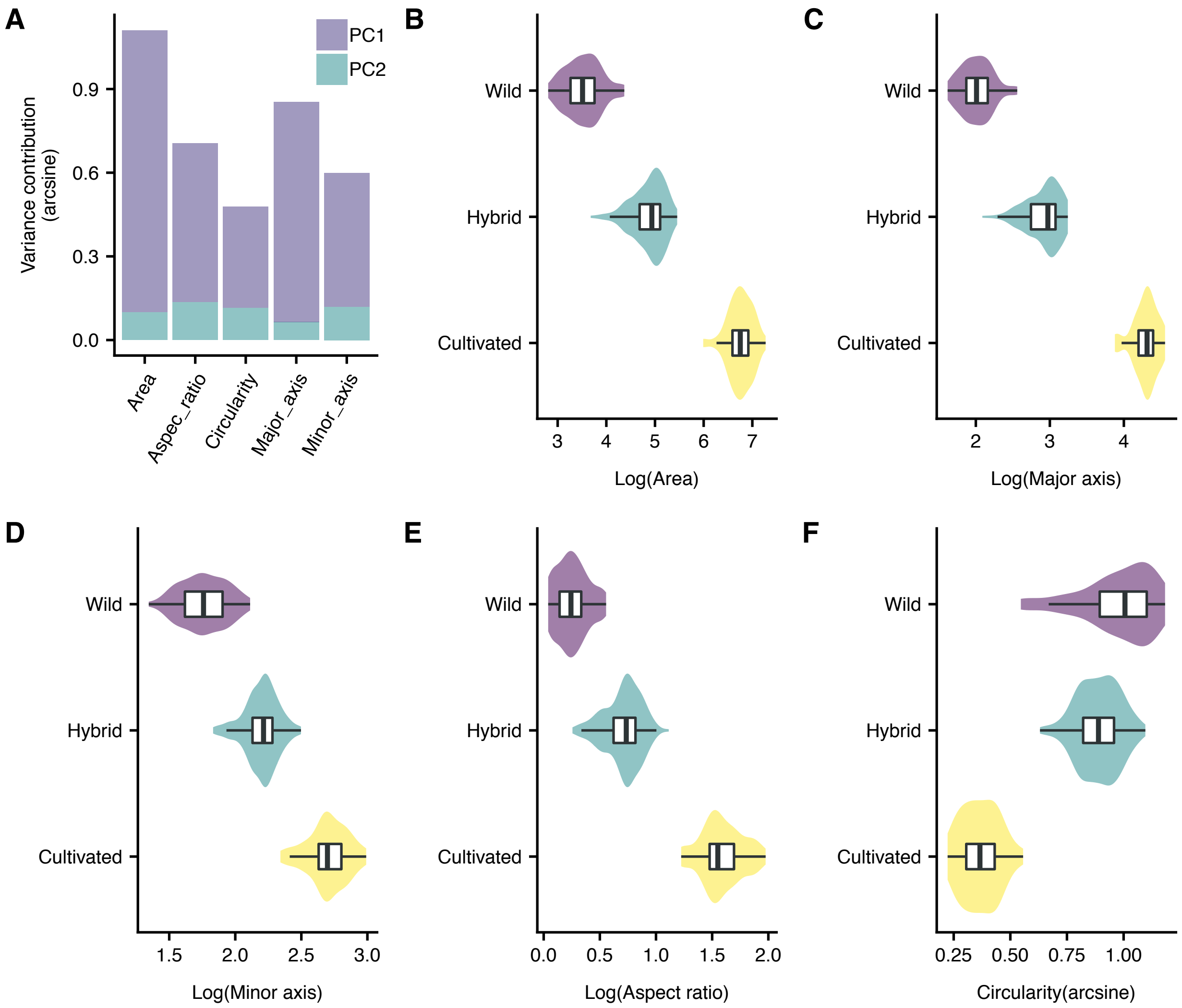

Figure S1. Morphometric analysis of fruit shape and size descriptors. A) Stacked bar plot showing the variance contributions of each morphometric descriptor to the principal components one and two. The variance contribution was arcsine-transformed for visualization purposes. B-F) Violin plots of individual phenotypic traits across the three genotypes.

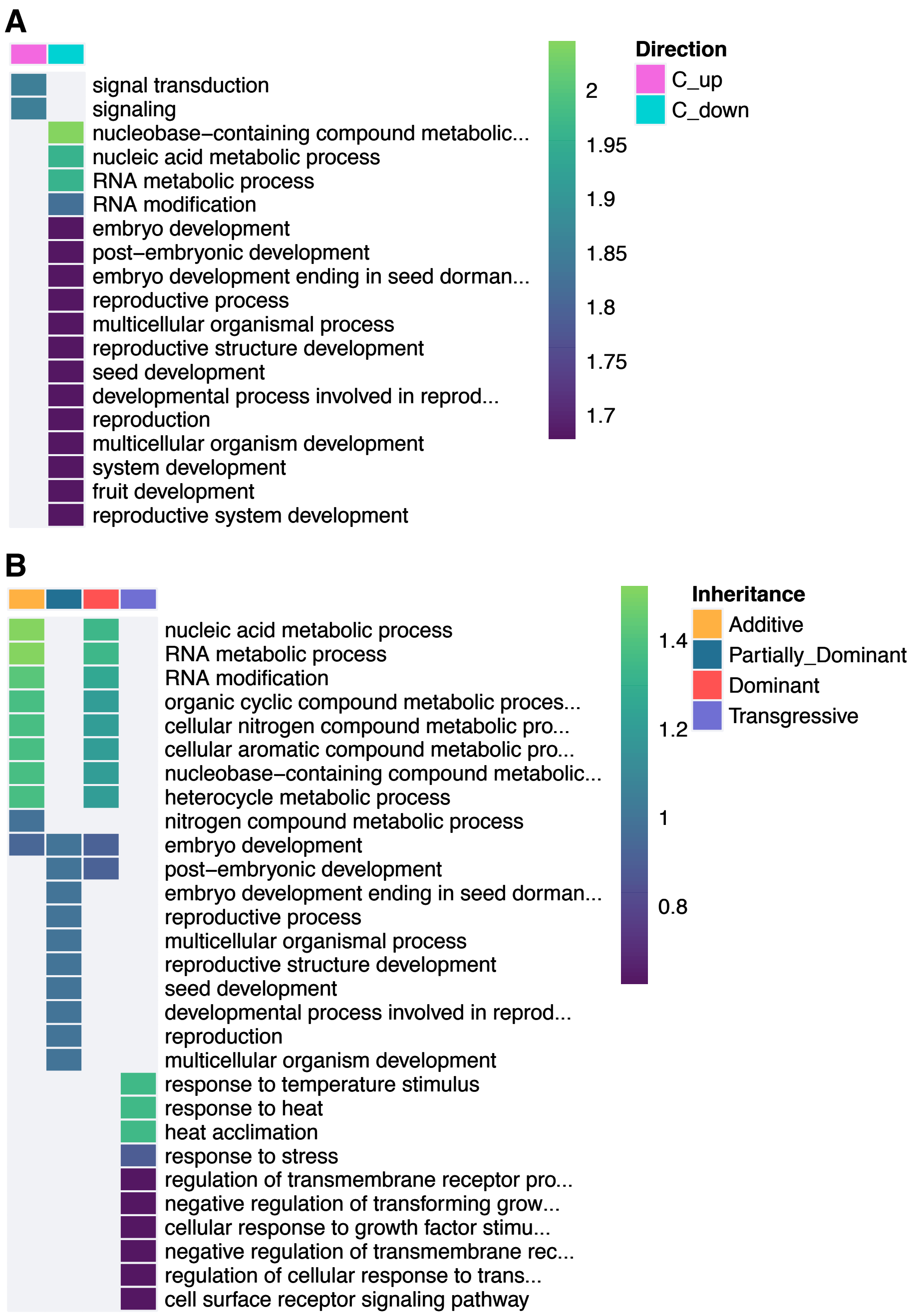

Figure S2. Heatmap of enriched gene ontology categories (biological processes) showing: A) The up- and down-regulation of the cultivated allele in F_0, and B) GO terms according to the mode of gene action (*k*).

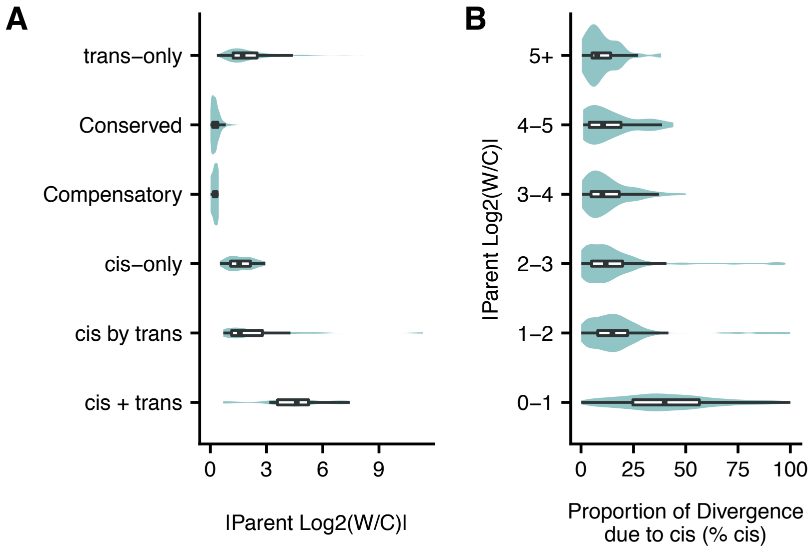

Figure S3. Features of *cis* and *trans*-regulated genes. A) Distribution of the effect sizes among parents for genes throughout all the categories of regulatory divergence. Genes with *cis*+*trans* produce the largest expression difference between parents. B) Proportion of divergence due to *cis* (% *cis*) in six different bins of expression divergence between parents, showing that genes have the least difference between parents also have the largest % *cis*.

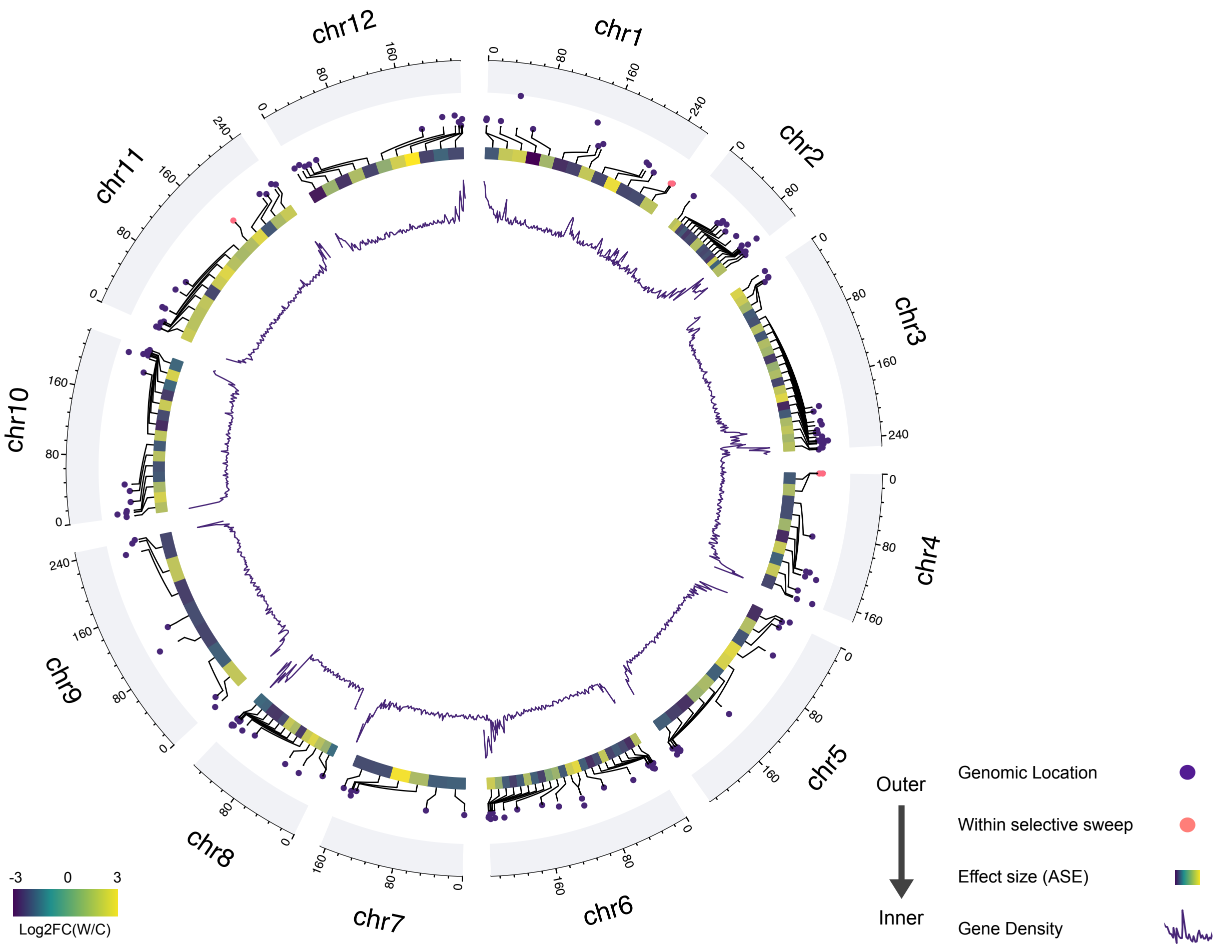

Figure S4. Circular representation of the twelve chili pepper chromosomes showing atributes of genes with *cis*-regulatory variation. The outermost track depicts the length of each chromosome in a Mb scale. The next track shows the genomic location and the -LogFDR of genes with significative ASE. Next, the effect size (Log_2 fold change) of differential expression between the wild and the cultivated allele F_0 is shown as a heatmap. The innermost track represents gene density as RNA-seq coverage in 2-Mb windows.

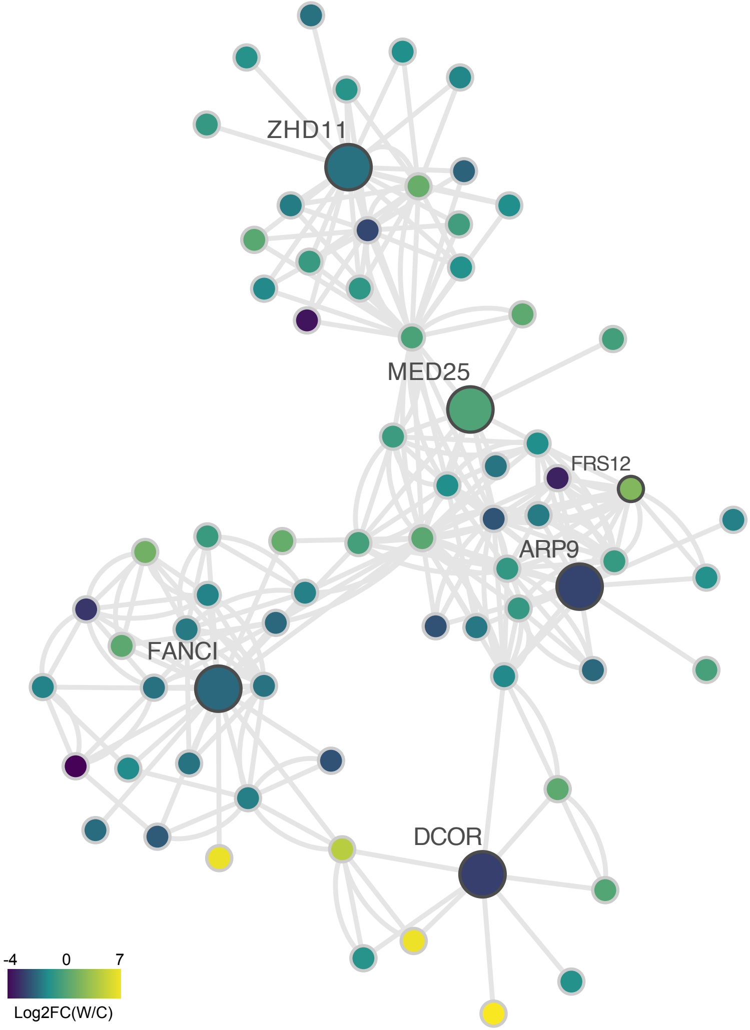

Figure S5. AracNe-based co-expression network representing putative interaction among the *cis* only candidates. The network was built by merging the individual networks of ZDH11, MED25, ARP9, DCOR FANCI and the FRS12 transcription factor. Color of nodes represent the Log2 fold change (W/C) in F_0 generation. The length of the edges represents the strength of the co-expression.
